# Lifetime environmental tobacco smoke exposure and the risk of chronic obstructive pulmonary disease

**DOI:** 10.1186/1476-069X-4-7

**Published:** 2005-05-12

**Authors:** Mark D Eisner, John Balmes, Patricia P Katz, Laura Trupin, Edward H Yelin, Paul D Blanc

**Affiliations:** 1 Department of Medicine, University of California, San Francisco, UCSF Box 0924, San Francisco, CA 94113-0924, USA; 2 Division of Occupational and Environmental Medicine, Department of Medicine, University of California, San Francisco, UCSF Box 0924, San Francisco, CA 94113-0924, USA; 3 Division of Pulmonary and Critical Care Medicine, Department of Medicine, University of California, San Francisco, UCSF Box 0924, San Francisco, CA 94113-0924, USA; 4 Institute for Health Policy Studies, University of California, San Francisco, UCSF Box 0920, San Francisco, CA 94113-0920, USA

**Keywords:** Pulmonary Disease, Chronic Obstructive, Chronic Bronchitis, Pulmonary Emphysema, Tobacco smoke pollution

## Abstract

**Background:**

Exposure to environmental tobacco smoke (ETS), which contains potent respiratory irritants, may lead to chronic airway inflammation and obstruction. Although ETS exposure appears to cause asthma in children and adults, its role in causing COPD has received limited attention in epidemiologic studies.

**Methods:**

Using data from a population-based sample of 2,113 U.S. adults aged 55 to 75 years, we examined the association between lifetime ETS exposure and the risk of developing COPD.

Participants were recruited from all 48 contiguous U.S. states by random digit dialing. Lifetime ETS exposure was ascertained by structured telephone interview. We used a standard epidemiologic approach to define COPD based on a self-reported physician diagnosis of chronic bronchitis, emphysema, or COPD.

**Results:**

Higher cumulative lifetime home and work exposure were associated with a greater risk of COPD. The highest quartile of lifetime home ETS exposure was associated with a greater risk of COPD, controlling for age, sex, race, personal smoking history, educational attainment, marital status, and occupational exposure to vapors, gas, dusts, or fumes during the longest held job (OR 1.55; 95% CI 1.09 to 2.21). The highest quartile of lifetime workplace ETS exposure was also related to a greater risk of COPD (OR 1.36; 95% CI 1.002 to 1.84). The population attributable fraction was 11% for the highest quartile of home ETS exposure and 7% for work exposure.

**Conclusion:**

ETS exposure may be an important cause of COPD. Consequently, public policies aimed at preventing public smoking may reduce the burden of COPD-related death and disability, both by reducing direct smoking and ETS exposure.

## Introduction

COPD is a common disease, affecting 5–10% of the population of North America and Europe [1-3]. During the past two decades, death and disability from COPD have continued to increase worldwide [1,4]. Although direct cigarette smoking is the major cause of COPD, up to two cases out of ten cannot be explained solely by direct smoking [5]. Environmental tobacco smoke (ETS) exposure, which appears to cause new cases of asthma, could also cause COPD [6-8]. Because it contains potent airway irritants, ETS could lead to chronic airway irritation, inflammation, and obstruction [6,9]. The role of ETS exposure in causing COPD, however, has received limited attention in epidemiologic studies [10,11]. Using data from a population-based sample of U.S. adults, we examined the association between lifetime ETS exposure and the risk of developing COPD.

## Methods

We used cross-sectional data from a cohort study of U.S. adults to elucidate the impact of lifetime ETS exposure on the risk of developing COPD. Initial recruitment and survey methods have been previously reported in detail [12]. The study was approved by the University of California, San Francisco Committee on Human Research. Briefly, 2,113 adults aged 55 to 75 years were recruited by random digit dialing among residents of the 48 contiguous U.S. states with random over-sampling of geographic areas that had the highest published COPD mortality rates [13]. The random "hot spot" sample was further enriched for additional subjects with COPD. The overall study participation rate was 53% among households with an eligible respondent present. Participants completed structured telephone interviews that included health history, work history, smoking, ETS exposure, and sociodemographic characteristics.

We used the standard epidemiologic approach to define COPD based on a self-reported physician diagnosis of chronic bronchitis, emphysema, or COPD [1,14,15]. During the telephone interview, subjects were asked whether they had ever received a physician's diagnosis of any of several chronic respiratory conditions. Those who reported physician diagnoses of chronic bronchitis or emphysema were considered to have COPD, along with those who specifically reported a physician diagnosis of COPD. We included respondents with COPD who had concomitant asthma because they clinically resemble persons with COPD alone [16].

We obtained spirometry reports from the physicians of a subgroup of 47 participants with COPD. The majority (89%) had airflow obstruction, as indicated by a FEV_1_/FVC ratio of less than 0.70 or an FEV_1 _less than 80% predicted. These physiologic data support the validity of our case definition for COPD.

We ascertained lifetime cumulative ETS exposure at home and work. Prenatal exposure was evaluated using the following survey item: "Did your mother smoke cigarettes when she was pregnant with you before you were born?" Subsequent home ETS exposure was assessed during childhood and adolescence using the following survey item: "Growing up until age 18, for how many years in total did you live in the same household with someone else who smoked tobacco products?" In addition, we measured home ETS exposure during adulthood: "Since age 18, for how many years in total have you lived in the same household with someone else who smoked tobacco products?" Cumulative lifetime home ETS exposure was calculated using the sum of both home exposures in years. Moreover, we ascertained cumulative lifetime work ETS exposure using the following item: "Thinking about all of the jobs you have had, for how many years of your employment have you been regularly exposed to another person's cigarette smoke inside your workplace?"

Direct personal cigarette smoking was evaluated using standard questions from the National Health Interview Survey [17]. Because workplace ETS exposure may be higher in occupations that involve exposure to other airway irritants and particulates, we also ascertained occupational exposure to vapors, gas, dusts, or fumes during the longest held job using a survey item developed for the European Community Respiratory Health Survey [18].

### Statistical analysis

Statistical analysis was conducted using SAS 8.2 (Cary, NC). Bivariate analysis was conducted using the unpaired t-test for continuous variables and likelihood ratio chi-square test for dichotomous variables. We examined the impact of lifelong cumulative ETS exposure and the risk of COPD. Based on the distribution of cumulative ETS exposure at home and work, we defined quartiles of exposure. For lifetime work exposure, the first quartile was zero years, so the first and second quartiles were collapsed as the referent group (otherwise the first and second quartile groups would both include zero values). We used logistic regression analysis to examine the relationship between each measure of ETS exposure and the risk of self-reported COPD. We used multivariate logistic regression analysis to control for factors that could confound the relation between ETS exposure and COPD, including past smoking history, age, sex, race-ethnicity, educational attainment, marital status, and occupational exposure to VGDF [12,19].

Consistent with the low prevalence of COPD among never smokers, there were too few never smokers with COPD (n = 75) to analyze this stratum separately. To address this issue, we controlled for the potential confounding effects of direct personal smoking in several ways. In the primary strategy, we included a history of ever smoking in the multivariate analysis. In an alternative analysis, we controlled for current smoking and ex-smoking as separate variables. There were no appreciable differences compared to primary analysis (this alternative analysis is not reported). We also restricted the multivariate analysis to subjects who reported no current smoking. There were no substantive differences compared to the primary analysis and these data are not reported. As an additional alternative analysis, we controlled for cumulative lifetime pack-years of smoking.

Both home and workplace ETS exposure independently contribute to lifetime cumulative ETS exposure. Consequently, workplace ETS exposure is not a confounder in the putative pathway between home ETS exposure and the development of COPD. Similarly, home ETS exposure does not operate as a confounder in the relationship between workplace ETS exposure and COPD onset. Based on these considerations, we used separate logistic regression analysis models to examine cumulative lifetime home and workplace ETS exposure, rather than including both exposures in the same model. In a secondary analysis, we examined home and work exposure in a mutually adjusted model.

Prenatal ETS exposure, which occurs via the placental circulation, may affect lung development and the subsequent risk of COPD by a different causal pathway than postnatal ETS exposure [21,22]. In fact, prenatal ETS exposure might be associated with both postnatal ETS exposure and the risk of COPD, confounding the relationship between postnatal ETS exposure and COPD. To address this possibility, we conducted an additional analysis to examine the independent relation between the two measures of cumulative lifetime ETS exposure (home and work), taking prenatal ETS exposure into account.

We also examined whether prenatal ETS exposure, occupational exposure to VGDF, or direct personal smoking modified the association between home and work ETS exposure and the risk of COPD. To accomplish this, interaction terms were evaluated in logistic regression models that included main effects for ETS exposure, the potential effect modifier, and personal smoking history. We evaluated significant statistical interactions for evidence of synergism or antagonism on an additive scale, which is more appropriate than a multiplicative scale for examining how two factors might biologically interact to produce disease. Using the odds ratio as an estimate of the relative risk, we used the formula OR-1 to calculate the relative excess risk [23,24]. If the relative excess risk for exposure to both factors together (e.g., direct smoking and ETS) is greater than the sum of the relative excess risks of each factor alone (e.g., smoking + ETS), a synergistic effect would be present. If the relative excess risk for exposure to both factors is less than the sum of the relative excess risks of each factor alone, antagonism would be present.

As a sensitivity analysis, we repeated the analysis using a more restrictive definition of COPD that included only those who specifically reported a diagnosis of emphysema or COPD, excluding those with chronic bronchitis alone.

We used the method of Greenland and Drescher to estimate the population attributable fraction from the multivariate logistic regression analysis controlling for personal smoking, sociodemographic factors, and occupational VGDF exposure [25]. This methodology provides a maximum likelihood estimator of the attributable fraction from the logistic model.

## Results

### Subject characteristics

The prevalence of COPD was 18% among the cohort of adults aged 55 to 75 years (95% CI 17 to 20%). Adults with COPD were more likely to be female and unmarried; they also had lower educational attainment than those without COPD (Table 1). A greater proportion of adults with COPD indicated ever smoking cigarettes compared to other members of the general population (81% vs. 56%), with substantially more current smokers (33 vs. 16%).

**Table 1:** Personal characteristics in a population-based sample of 2,113 U.S. adults aged 55 to 75 years

Characteristic	COPD (n = 386)	No COPD (n = 1,727)	P value
Age (years)	64.3 (6.2)	63.9 (6.1)	0.24
Gender (female)	246 (64%)	961 (56%)	0.004
Race-ethnicity (white)	337 (87%)	1495 (87%)	0.70
Married or cohabitating	186 (48%)	1080 (63%)	<0.0001
Educational attainment			<0.0001
High school degree	207 (54%)	709 (41%)	
Some college	105 (27%)	529 (31%)	
College or graduate degree	74 (19%)	489 (28%)	
Cigarette smoking			<0.0001
Current smoker	127 (33%)	279 (16%)	
Past smoker	184 (48%)	685 (40%)	
Never smoker	75 (19%)	763 (44%)	

Proportions are column proportions (of those with and without COPD)

P-values from unpaired t-test (age) and likelihood ratio chi-square test

### ETS exposure

The prevalence of previous prenatal ETS exposure was higher among adults with COPD than those without the condition (8.3% vs. 5.6%, p = 0.051) (Table 2). Moreover, the lifetime prevalence of any subsequent lifetime home or workplace ETS exposure was higher among those with COPD than among those without COPD (89% vs. 82%, p = 0.0004 and 67% vs. 60%, p = 0.012, respectively). Persons with COPD were also more likely to have a higher cumulative lifetime exposure to ETS, with a greater proportion of the COPD group having the highest quartile of home and workplace exposure than those without the condition (41% vs. 22% and 34% vs. 24%, respectively) (Table 2).

**Table 2:** Lifetime cumulative ETS exposure in a population-based sample of adults aged 55–75 years

Source of ETS exposure	COPD (n = 386)	No COPD (n = 1,727)	P value
Prenatal ETS*	32 (8.3%)	96 (5.6%)	0.051
Cumulative lifetime home ETS			<0.0001
Quartile 1 (0–9 yrs)	70 (18%)	443 (26%)	
Quartile 2 (10–21 yrs)	72 (19%)	461 (27%)	
Quartile 3 (22–41 yrs)	86 (22%)	451 (26%)	
Quartile 4 (≥42 yrs)	158 (41%)	372 (22%)	
Cumulative lifetime work ETS			0.0002
Quartile 1 & 2 (0–5 yrs)^†^	163 (42%)	881 (51%)	
Quartile 3 (6–22 yrs)	93 (24%)	440 (25%)	
Quartile 4 (≥23 yrs)	130 (34%)	406 (24%)	

Proportions are column proportions (of those with and without COPD)

P-values from the likelihood ratio chi-square test

*Mother smoked during pregnancy

^†^First and second quartile were both zero, so they were combined into one group.

### ETS exposure and the risk of COPD

Maternal smoking during pregnancy was not statistically associated with a higher risk of COPD, after controlling for personal smoking and sociodemographic covariates (OR 1.41; 95% CI 0.90 to 2.21) (Table 3). Higher cumulative lifetime home and work exposure were, however, related to a greater risk of developing COPD. The highest quartile of home ETS exposure was associated with a greater risk of COPD (OR 1.68; 95% CI 1.19 to 2.38). The highest quartile of workplace ETS exposure was also associated with a higher risk of COPD (OR 1.60; 95% CI 1.20 to 2.14). After controlling for workplace VGDF exposure, home and workplace ETS exposure remained associated with the risk of COPD (OR 1.55; 95% CI 1.09 to 2.21 and OR 1.36; 95% CI 1.002 to 1.84, respectively). Based on this most adjusted analysis, the population attributable fraction was 11% for the highest quartile of home ETS exposure and 7% for work exposure.

**Table 3:** Lifetime cumulative ETS exposure and the risk of COPD in a population based sample of 2,113 U.S. adults aged 55 to 75 years

Source of exposure	Level of exposure	Risk of COPD (unadjusted)	Risk of COPD, controlling for smoking	Risk of COPD, controlling for smoking, sociodemographic indicators^†^	Risk of COPD, controlling for smoking, sociodemographic indicators, and workplace VGDF^†^
		OR (95% CI)	OR (95% CI)	OR (95% CI)	OR (95% CI)
Prenatal	Mother smoked in pregnancy	1.54 (1.01 to 2.33)	1.32 (0.86 to 2.01)	1.41 (0.90 to 2.21)	1.36 (0.86 to 2.14)
					
Home	1st quartile (0–9 yrs)	1.0 (referent)	1.0 (referent)	1.0 (referent)	1.0 (referent)
					
	2nd quartile (10–21 yrs)	0.99 (0.69 to 1.41)	0.88 (0.61 to 1.25)	0.92 (0.64 to 1.34)	0.90 (0.62 to 1.31)
					
	3rd quartile (22–41 yrs)	1.21 (0.86 to 1.70)	0.95 (0.67 to 1.35)	0.95 (0.66 to 1.37)	0.94 (0.65 to 1.36)
					
	4th quartile (≥42 yrs)	2.69 (1.97 to 3.68)	1.88 (1.35 to 2.61)	1.68 (1.19 to 2.38)	1.55 (1.09 to 2.21)
					
Work	1st & 2nd quartile (0–5 yrs)*	1.0 (referent)	1.0 (referent)	1.0 (referent)	1.0 (referent)
					
	3rd quartile (6–22 yrs)	1.14 (0.86 to 1.51)	0.97 (0.73 to 1.30)	1.02 (0.76 to 1.38)	0.91 (0.67 to 1.24)
					
	4th quartile (≥23 yrs)	1.73 (1.34 to 2.24)	1.34 (1.02 to 1.75)	1.60 (1.20 to 2.14)	1.36 (1.002 to 1.84)

VGDF = self-reported exposure to vapors, gas, dust, or fumes during longest held job

*First and second quartile were both zero, so they were combined as the referent group (otherwise both lower categories would include zero values)

^†^Multivariate logistic regression analysis controlling for age, sex, race, smoking history, educational attainment, and marital status. Each source of ETS exposure was evaluated in a separate logistic regression model.

We also examined the independent relationship between cumulative lifetime ETS exposure and COPD, controlling for prenatal ETS exposure. The highest quartiles of home and workplace ETS exposure were associated with a greater risk of COPD, controlling for personal smoking, sociodemographic factors, occupational VGDF exposure, and prenatal ETS exposure (OR 1.64; 95% CI 1.16 to 2.33 and OR 1.59; 95% CI 1.19 to 2.13, respectively).

There was no evidence that prenatal ETS modified the relationship between ETS exposure and the risk of COPD (p values for interaction with home ETS = 0.59 and work ETS = 0.86). There was also no indication that occupational VGDF exposure modified the association between ETS exposure at home or work and the risk of COPD (p for interaction = 0.94 and 0.14, respectively).

There was evidence of a statistical interaction between home ETS exposure and a history of ever smoking for the risk of COPD (p = 0.041), whereas there was no interaction for workplace ETS exposure (p = 0.17). The relative excess risk for the highest quartile of home ETS exposure among never smokers was 0.88. The relative excess risk for personal smoking among those with little or no home ETS exposure (i.e., first quartile) was 1.83. The relative excess risk for combined high level home ETS exposure and personal smoking was 4.32, which exceeded the sum of the relative excess risks for high level home ETS and personal smoking (i.e., 4.32 > 0.88 + 1.83 or 4.32 > 2.71). These results are consistent with a synergistic effect of home ETS and smoking for COPD risk. In addition, these results underscore the risk conferred by home ETS exposure among never smokers (i.e., excess relative risk of 0.88).

### ETS exposure and the risk of COPD – secondary analyses

In a secondary analysis, we controlled for personal direct smoking history using cumulative lifetime pack-years of smoking. The highest quartile of home and workplace exposure were associated with a greater risk of COPD, controlling for pack-years of personal smoking and sociodemographic characteristics (OR 1.62; 95R CI 1.14 to 2.30 and OR 1.48; 95% CI 1.10 to 1.99, respectively). After controlling for workplace VGDF exposure, home and workplace ETS exposure were still related to a greater risk of COPD (OR 1.49; 95% CI 1.04 to 2.12 and OR 1.24; 95% CI 0.91 to 1.69, respectively). In the latter analysis, the confidence interval for workplace exposure widened and did not exclude no relationship.

In other alternative analyses, we also examined lifetime cumulative home and work ETS exposure in the same model (mutually adjusted model). The highest quartile of home (1.55; 95% CI 1.09 to 2.21) and workplace exposure (OR 1.46; 95% CI 1.08 to 1.96) were associated with a greater risk of COPD, controlling for smoking history and sociodemographic characteristics.

### Emphysema or COPD subgroup

In a sensitivity analysis, we examined the subgroup of subjects who reported emphysema or COPD (n = 189), excluding those who reported chronic bronchitis alone (n = 194). Greater cumulative lifetime home and work ETS exposure remained associated with a greater risk of COPD after controlling for all covariates (OR 2.38 for highest quartile; 95% CI 1.42 to 3.90 and OR 1.79; 95% CI 1.21 to 2.65).

## Discussion

ETS exposure, both at home and work, was associated with a greater risk of COPD in this population-based study of older adults, even after taking personal smoking history into account. On a population level, approximately 1 in 11 cases of COPD may be attributed, at least in part, to home ETS exposure; 1 in 15 cases may be attributable to workplace ETS exposure.

The previous epidemiologic literature, albeit limited, supports an association between ETS exposure and COPD. A cross-sectional population-based study from Switzerland found a relationship between self-reported ETS exposure during the past 12 months and a higher risk of chronic bronchitis symptoms [26]. A case-control study demonstrated that self-reported ETS exposure was associated with obstructive respiratory disease, defined as asthma, chronic bronchitis, or emphysema [27]. Reports from the Adventist Health Study of Smog (AHSMOG) indicated a relationship between self-reported ETS exposure and a greater risk of "airway obstructive disease" (asthma, chronic bronchitis, or emphysema), chronic bronchitis symptoms, and airway obstruction by pulmonary function testing [28,29]. These studies are limited by the lack of a comprehensive and specific definition of COPD (i.e., includes chronic bronchitis, emphysema, and COPD but not asthma), the absence of cumulative lifetime ETS exposure data, and the omission of other occupational exposures that could be correlated with ETS exposure.

The results suggest that home ETS exposure and personal smoking may act synergistically to increase the risk of COPD. There are several possible biological mechanisms that could account for this synergistic action. ETS contains potent respiratory irritants, such as formaldehyde and acrolein, which could directly irritate the airways and exacerbate smoking-related airflow obstruction. Both ETS and direct smoking may increase airway permeability, causing increased IgE levels and enhanced allergic sensitization to airborne antigens [30,31]. By this and other mechanisms, ETS and cigarette smoking could act to increase airway inflammation. Other possible mechanisms are combined effects of smoking and ETS on bronchial hyperresponsiveness [32]. Further experimental work will be necessary to elucidate the apparent synergy between ETS exposure and direct smoking.

Our results suggest that the highest quartiles of home and work ETS exposure were associated with a greater risk of COPD. Is it therefore possible to conclude that lower levels of ETS exposure are "safe" in terms of obstructive lung disease? We believe that our data do not suggest a "safe" level of ETS exposure. Based on our results, the 95% confidence intervals for the lower exposure quartiles are compatible with a substantially increased risk of COPD. Moreover, we have previously shown that very low levels of ETS exposure can exacerbate adult asthma [33]. We have also shown that moderate levels of ETS exposure are associated with impaired pulmonary function [34]. Taken together, these results indicate that even low-to-moderate levels of ETS exposure may have deleterious effects on airway function and obstructive lung disease.

We used the standard epidemiologic definition of COPD, based on a self-reported physician diagnosis of chronic bronchitis, emphysema, or COPD [1,14,15]. This survey-based approach enabled us to evaluate a population-based sample of adults who resided throughout the continental United States, which ensured generalizable results. On logistical grounds, conducting spirometry among subjects who reside thousands of miles apart would be highly difficult, if not impossible. The use of self-reported physician-diagnosis, however, may have resulted in some misclassification of disease status. Previous work indicated that a similar survey-based definition of COPD had a high positive predictive value (78%) when validated using a blinded medical record review that included spirometry and radiographic studies [35]. Other work confirmed that a self-reported history of COPD is a strong predictor of airflow obstruction [36]. In the subset of our participants with COPD who had available spirometry data, the prevalence of airflow obstruction was very high (89%). In addition, the high prevalence of lifetime smoking in our study, which was more than 80%, supports the diagnosis of COPD. The prevalence of COPD in our sample (18%) was also similar to that reported in two other population-based studies conducted in the United States [1]. Furthermore, reanalysis of our data using a more restrictive definition of COPD that excluded chronic bronchitis did not appreciably affect the results. In sum, misclassification of COPD is not likely to bias our results; if present, such bias would likely be non-differential with respect to ETS exposure and reduce effect estimates towards the null value.

Lifetime cumulative ETS exposure was ascertained by self-report, which could have resulted in exposure misclassification. Previous studies have found moderate correlations between self-reported ETS exposure and biomarker levels (e.g., cotinine) or direct personal exposure monitoring (e.g., nicotine) [33,37-42]. We cannot, however, exclude some systematic misclassification of ETS exposure. For example, persons with COPD, because they have respiratory symptoms, could be more likely to remember and report ETS exposure, upwardly biasing the effect estimates. Because our focus was on lifetime ETS exposure, there is no other available ETS exposure methodology. Cotinine level, the most widely used biomarker for ETS exposure, reflects exposure during the past 1–2 days [42]. Direct exposure monitoring, such as the personal nicotine badge, can only be used for brief periods of up to several weeks [33,43]. Consequently, the only feasible method for lifetime ETS exposure is survey-based.

Because smoking is the dominant risk factor for COPD, we cannot completely exclude some residual confounding by smoking. There were too few never smokers with COPD (n = 75) to restrict the overall analysis to never smokers. To address this issue, we controlled for personal smoking history in multivariate analysis, defined as ever smoking or current / past smoking. The multivariate analysis was also restricted to non-current smokers, yielding essentially the same results. We also controlled for cumulative lifetime pack-years of smoking in additional analyses, which continued to show highly significant results for home ETS exposure, but slightly attenuated findings for workplace ETS after VGDF exposure was also controlled. The interaction analysis also supported the elevated risk for home ETS exposure among never smokers. In sum, the results do not indicate that the results can be explained by residual confounding by direct personal smoking history.

## Conclusion

COPD is a leading cause of death and disability among middle-aged adults in developed nations [1,4,44]. Adults with COPD have a 10-fold higher risk of disability compared to members of the general population [16]. Although cigarette smoking is the dominant cause of COPD, other factors, such as occupational exposures, appear to contribute to disease causation [12, 45]. Based on our results, we believe that ETS exposure may also be an important cause of COPD. Consequently, public policies aimed at preventing public smoking may reduce the burden of COPD-related death and disability, both by reducing direct smoking and ETS exposure.

## Abbreviations

ETS = environmental tobacco smoke; COPD = chronic obstructive pulmonary disease

## Competing interests

The author(s) declare that they have no competing interests.

## Authors' contributions

MDE conceived the study, performed the analysis, and wrote the paper; JB participated in the design and analysis and helped draft the manuscript; Laura Trupin participated in the analysis and drafting of the manuscript; PK participated in the design and drafting of the manuscript; EH participated in the analysis, interpretation of data, and drafting of the manuscript; PB initiated the cohort study, participated in the design, analysis, and drafting of the manuscript.

## References

[B1] Mannino DM, Homa DM, Akinbami LJ, Ford ES, Redd SC (2002). Chronic obstructive pulmonary disease surveillance--United States, 1971-2000. MMWR Surveill Summ.

[B2] Halbert RJ, Isonaka S, George D, Iqbal A (2003). Interpreting COPD prevalence estimates: what is the true burden of disease?. Chest.

[B3] Rennard S, Decramer M, Calverley PM, Pride NB, Soriano JB, Vermeire PA, Vestbo J (2002). Impact of COPD in North America and Europe in 2000: subjects' perspective of Confronting COPD International Survey. Eur Respir J.

[B4] Murray CJ, Lopez AD (1997). Alternative projections of mortality and disability by cause 1990-2020: Global Burden of Disease Study. Lancet.

[B5] U.S. Department of Health EW (1971). The Health Consequences of Smoking: a Report to the Surgeon General.

[B6] California Environmental Protection Agency (1997). Health Effects of Exposure to Environmental Tobacco Smoke.

[B7] Eisner MD (2002). Environmental tobacco smoke and adult asthma. Clin Chest Med.

[B8] Jaakkola MS, Piipari R, Jaakkola N, Jaakkola JJ (2003). Environmental tobacco smoke and adult-onset asthma: a population-based incident case-control study. Am J Public Health.

[B9] Nikula KJ, Green FH (2000). Animal models of chronic bronchitis and their relevance to studies of particle-induced disease. Inhal Toxicol.

[B10] Jaakkola MS, Jaakkola JJ (2002). Effects of environmental tobacco smoke on the respiratory health of adults. Scand J Work Environ Health.

[B11] Coultas DB (1998). Health effects of passive smoking. 8. Passive smoking and risk of adult asthma and COPD: an update. Thorax.

[B12] Trupin L, Earnest G, San Pedro M, Balmes JR, Eisner MD, Yelin E, Katz PP, Blanc PD (2003). The occupational burden of chronic obstructive pulmonary disease. Eur Respir J.

[B13] Kim J (1998). Atlas of Respiratory Disease Mortality, United States: 1982-1993..

[B14] Sin DD, Stafinski T, Ng YC, Bell NR, Jacobs P (2002). The impact of chronic obstructive pulmonary disease on work loss in the United States. Am J Respir Crit Care Med.

[B15] Mannino DM (2002). COPD: epidemiology, prevalence, morbidity and mortality, and disease heterogeneity. Chest.

[B16] Eisner MD, Yelin EH, Trupin L, Blanc PD (2002). The Influence of Chronic Respiratory Conditions on Health Status and Work Disability. Am J Public Health.

[B17] (2002). Cigarette smoking among adults--United States, 2000. MMWR Morb Mortal Wkly Rep.

[B18] United Medical and Dental Schools of Guy's and St Thomas' Hospital Department of Public Health Medicine (1993). Protocol for the European Respiratory Community Health Survey.

[B19] Iribarren C, Friedman GD, Klatsky AL, Eisner MD (2001). Exposure to environmental tobacco smoke: association with personal characteristics and self reported health conditions. J Epidemiol Community Health.

[B20] Tager IB, Ngo L, Hanrahan JP (1995). Maternal smoking during pregnancy. Effects on lung function during the first 18 months of life. Am J Respir Crit Care Med.

[B21] Hanrahan JP, Tager IB, Segal MR, Tosteson TD, Castile RG, Van Vunakis H, Weiss ST, Speizer FE (1992). The effect of maternal smoking during pregnancy on early infant lung function. Am Rev Respir Dis.

[B22] Rothman K, Keller A (1972). The effect of joint exposure to alcohol and tobacco on risk of cancer of the mouth and pharynx. J Chron Dis.

[B23] Cole P, MacMahon B (1971). Attributable risk percent in case control studies. Brit J Prev Soc Med.

[B24] Greenland S, Drescher K (1993). Maximum likelihood estimation of the attributable fraction from logistic models. Biometrics.

[B25] Leuenberger P, Schwartz J, Ackermann-Liebrich U, Blaser K, Bolognini G, Bongard JP, Brandli O, Braun P, Bron C, Brutsche M (1994). Passive smoking exposure in adults and chronic respiratory symptoms (SAPALDIA Study). Swiss Study on Air Pollution and Lung Diseases in Adults, SAPALDIA Team [see comments]. Am J Respir Crit Care Med.

[B26] Dayal HH, Khuder S, Sharrar R, Trieff N (1994). Passive smoking in obstructive respiratory disease in an industrialized urban population. Environ Res.

[B27] Robbins AS, Abbey DE, Lebowitz MD (1993). Passive smoking and chronic respiratory disease symptoms in non-smoking adults. Int J Epidemiol.

[B28] Berglund DJ, Abbey DE, Lebowitz MD, Knutsen SF, McDonnell WF (1999). Respiratory symptoms and pulmonary function in an elderly nonsmoking population [see comments]. Chest.

[B29] Sapigni T, Biavati P, Simoni M, Viegi G, Baldacci S, Carrozzi L, Modena P, Pedreschi M, Vellutini M, Paoletti P (1998). The Po River Delta Respiratory Epidemiological Survey: an analysis of factors related to level of total serum IgE. Eur Respir J.

[B30] Oryszczyn MP, Annesi-Maesano I, Charpin D, Paty E, Maccario J, Kauffmann F (2000). Relationships of active and passive smoking to total IgE in adults of the Epidemiological Study of the Genetics and Environment of Asthma, Bronchial Hyperresponsiveness, and Atopy (EGEA). Am J Respir Crit Care Med.

[B31] Janson C, Chinn S, Jarvis D, Zock JP, Toren K, Burney P (2001). Effect of passive smoking on respiratory symptoms, bronchial responsiveness, lung function, and total serum IgE in the European Community Respiratory Health Survey: a cross-sectional study. Lancet.

[B32] Eisner MD, Katz PP, Yelin EH, Hammond SK, Blanc PD (2001). Measurement of environmental tobacco smoke exposure among adults with asthma. Environmental Health Perspectives.

[B33] Eisner MD (2002). Environmental tobacco smoke exposure and pulmonary function among adults in NHANES III: impact on the general population and adults with current asthma. Environ Health Perspect.

[B34] Barr RG, Herbstman J, Speizer FE, Camargo CAJ (2002). Validation of self-reported chronic obstructive pulmonary disease in a cohort study of nurses. Am J Epidemiol.

[B35] Straus SE, McAlister FA, Sackett DL, Deeks JJ (2002). Accuracy of history, wheezing, and forced expiratory time in the diagnosis of chronic obstructive pulmonary disease. J Gen Intern Med.

[B36] Pirkle JL, Flegal KM, Bernert JT, Brody DJ, Etzel RA, Maurer KR (1996). Exposure of the US population to environmental tobacco smoke: the Third National Health and Nutrition Examination Survey, 1988 to 1991 [see comments]. Jama.

[B37] Delfino RJ, Ernst P, Jaakkola MS, Solomon S, Becklake MR (1993). Questionnaire assessments of recent exposure to environmental tobacco smoke in relation to salivary cotinine. Eur Respir J.

[B38] Emmons KM, Abrams DB, Marshall R, Marcus BH, Kane M, Novotny TE, Etzel RA (1994). An evaluation of the relationship between self-report and biochemical measures of environmental tobacco smoke exposure. Prev Med.

[B39] Coultas DB, Peake GT, Samet JM (1989). Questionnaire assessment of lifetime and recent exposure to environmental tobacco smoke. Am J Epidemiol.

[B40] Jaakkola MS, Jaakkola JJ (1997). Assessment of exposure to environmental tobacco smoke. Eur Respir J.

[B41] Benowitz NL (1999). Biomarkers of environmental tobacco smoke exposure. Environ Health Perspect.

[B42] Leaderer BP, Hammond SK (1987). A diffusion monitor to measure exposure to passive smoking.. Environmental Science Technology.

[B43] Verbrugge LM, Patrick DL (1995). Seven chronic conditions: their impact on US adults' activity levels and use of medical services. Am J Public Health.

[B44] Balmes J, Becklake M, Blanc P, Henneberger P, Kreiss K, Mapp C, Milton D, Schwartz D, Toren K, Viegi G (2003). American Thoracic Society Statement: Occupational contribution to the burden of airway disease. Am J Respir Crit Care Med.

